# Oncolytic Viral Therapy for Mesothelioma

**DOI:** 10.3389/fonc.2017.00179

**Published:** 2017-08-24

**Authors:** Daniel F. Pease, Robert A. Kratzke

**Affiliations:** ^1^Hematology-Oncology-Transplant, University of Minnesota, Minneapolis, MN, United States

**Keywords:** mesothelioma, oncolytic, virotherapy, novel, measles virus, adenovirus, herpes simplex virus type 1, vaccinia virus

## Abstract

The limited effectiveness of conventional therapy for malignant pleural mesothelioma demands innovative approaches to this difficult disease. Even with aggressive multimodality treatment of surgery, radiation, and/or chemotherapy, the median survival is only 1–2 years depending on stage and histology. Oncolytic viral therapy has emerged in the last several decades as a rapidly advancing field of immunotherapy studied in a wide spectrum of malignancies. Mesothelioma makes an ideal candidate for studying oncolysis given the frequently localized pattern of growth and pleural location providing access to direct intratumoral injection of virus. Therefore, despite being a relatively uncommon disease, the multitude of viral studies for mesothelioma can provide insight for applying such therapy to other malignancies. This article will begin with a review of the general principles of oncolytic therapy focusing on antitumor efficacy, tumor selectivity, and immune system activation. The second half of this review will detail results of preclinical models and human studies for oncolytic virotherapy in mesothelioma.

## Introduction: Standard Therapy for Mesothelioma

Mesothelioma is an uncommon malignancy of the parietal and visceral mesothelium, with about 3,300 new cases each year in the United States (1). Malignant pleural mesothelioma (MPM) accounts for 90% of cases, as inhalation asbestos exposure is the major risk factor. Most of the remaining cases arise from the peritoneum, with only 1–2% of cases occurring in the pericardium or tunica vaginalis testis (2). In the western world, incidence peaked in the early 21st century and has since leveled off in the US, while in Europe estimates are for a decrease in new cases (3–5). This is the result of concerted efforts over the last several decades to reduce asbestos exposure. Unfortunately, less developed countries that are slower to control asbestos exposure likely will continue to see an increase in incidence because of the prolonged latency period of at least 20 years before development of mesothelioma (6, 7).

The typical presenting symptoms of MPM are non-specific and include shortness of breath, chest pain, and weight loss. Characteristic findings on chest imaging are pleural abnormalities such as a unilateral effusion, calcified plaques, thickening, or masses (8). Diagnosis often requires a full-thickness pleural biopsy *via* pleuroscopy or video-assisted thoracoscopy. Pleural fluid cytology, although more easily obtained, is usually not sufficient. Even with adequate tissue, the pathologic evaluation can be challenging as mesothelioma is not frequently seen in most centers and has a number of different subtypes—epithelioid, sarcomatoid, biphasic—that must be differentiated from reactive processes in the pleura (9).

The management of mesothelioma is to the extent possible multimodality strategy incorporating chemotherapy, surgery, and/or radiation. The initial step is evaluating whether the disease is surgically resectable, with the goal of macroscopic complete resection. The two main surgical techniques are extrapleural pneumonectomy (EPP) or the less radical pleurectomy/decortication (P/D). Comparisons of EPP and P/D are limited to observational studies, with the largest cohort showing a survival advantage to P/D (10). A recent meta-analysis found lower short-term mortality for P/D, although the 2-year survival was not significantly different than EPP (11). In the absence of randomized trial data, the surgical approach is determined on a patient-specific basis.

Chemotherapy for MPM is recommended for all patients undergoing active therapy, with either cisplatin or carboplatin combined with pemetrexed as the standard of care. In patients not eligible for surgical resection, cisplatin/pemetrexed was shown to have a superior median overall survival compared to cisplatin monotherapy of 12.1 vs. 9.3 months (12). Carboplatin is equally efficacious to cisplatin in combination with pemetrexed, providing an alternative for older patients and those with borderline renal function (13). The addition of bevacizumab to cisplatin/pemetrexed may offer further benefit, pushing median overall survival to 18 months (14). For those patients having surgical resection, chemotherapy is given either preoperatively or postoperatively with no studies comparing the two approaches.

The role of radiation therapy is less clear, with most studies evaluating its use in the postoperative setting to reduce the risk of local recurrence (15). Trimodality therapy of preoperative chemotherapy, surgical resection, and postoperative radiation has been evaluated in small studies with variable success (16, 17). More detailed reviews of standard therapy for pleural and peritoneal mesothelioma are available elsewhere (8, 18, 19).

Despite the application of multimodality therapy to MPM, most patients are candidates for only palliative chemotherapy and have a median overall survival of 1–2 years (20). These limitations in current treatment highlight the importance of investigational therapies that may improve the prognosis of an otherwise highly fatal disease. This review will focus on the use of oncolytic viral therapy for mesothelioma.

## The Principles of Oncolytic Viral Therapy

### Background

The fact that viruses may inflict damage not only in healthy human tissue but also in tumor cells was first observed in the early 21st century (21). The first formal studies utilizing viruses as anticancer therapy were performed in the 1950s and documented transient tumor response in a small number of patients (22–24). However, these intriguing early results were tempered by technical and methodological constraints, and investigation declined for the next few decades (25).

A renewed interest began in the late 20th century as scientific advances in virology and molecular genetics allowed greater viral manipulation and the potential for increased efficacy (26). Many viruses have now been studied in this context, including adenovirus, herpes simplex virus (HSV), vaccinia, measles virus, and others, applied against a number of malignancies such as glioma, breast, head and neck, and lung (27–30). In 2015, a genetically modified HSV type 1 (HSV-1) (T-VEC) became the first FDA-approved oncolytic viral therapy, for use against melanoma (31).

**Figure 1:**
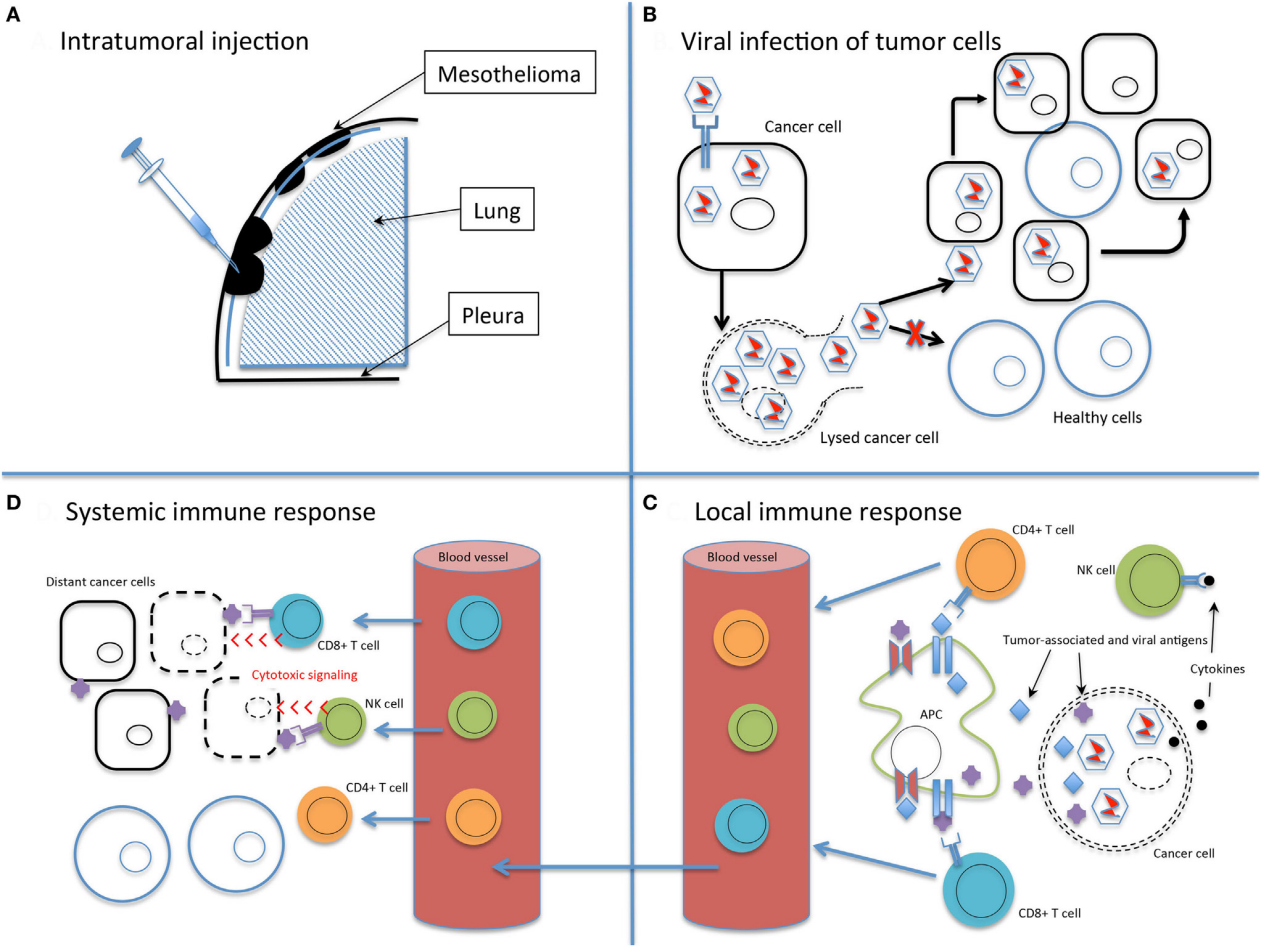
The basic principles of oncolytic virotherapy. **(A)** Administration is most commonly *via* direct intratumoral injection rather than systemic intravenous route to avoid viral inactivation in the bloodstream and minimize off-target infection. The pleural location of mesothelioma is particularly amenable to direct injection. **(B)** Viral infection of cancer cells, followed by replication, leads to cell lysis and dissemination of infection. The use of non-replicating viruses results in lysis to a lesser extent than replicating viruses. Acquired defects of the cancer cells and engineered modifications of the viral genome drive infection selectively toward cancer cells. **(C)** Viral infection and lysis exposes tumor-associated and viral antigens to the immune system. Antigen-presenting cells process these novel antigens *via* the major histocompatibility complex for presentation to CD4+ and CD8+ T cells. Cytokine release attracts NK cells. Local tumor cell death is augmented by the immune response. **(D)** Activated T and NK cells circulate throughout the body and recognize distant tumor cells that express the previously uncovered tumor-associated antigens. Note that the systemic immune response is not dependent on viral oncolysis.

The ideal oncolytic viral therapy is based on three basic principles (32, 33): (1) antitumor efficacy, the ability to directly infect and lyse tumor cells; (2) tumor selectivity, to preferentially infect tumor cells and minimize toxicity of infection to healthy tissue; and perhaps most importantly, (3) stimulation of the immune system, to provoke an antitumor response that will amplify the viral-directed cell death and provide ongoing tumor cell killing (Figure 1). Genetically engineering viruses to optimize tumor cell toxicity and selectivity has found success, while attaining a sustained immunotherapeutic response has proven a more difficult task.

### Antitumor Efficacy

The concern for toxicity of wild-type viruses led to the first recombinant viruses being engineered as replication-incompetent strains, with the goal of delivering gene therapy but not necessarily propagation of viral infection (34). The development of techniques to enhance viral selectivity for tumor cells allowed a shift back toward using replication-competent oncolytic viruses. These virulent models allow the natural viral mechanisms to infect, replicate, and lyse tumor cells. As virions are released from lysed tumor cells, the infection spreads within the local tumor mass (33). This potentiates tumor cell killing compared to the initial input dose of viral particles and may lead to a more robust antitumor response from the immune system (32). Gene therapy with replication-incompetent viruses has a bystander effect that also may amplify cell death in the local tumor environment, although to a lesser extent than actively replicating viruses (25).

The mechanisms of tumor cell killing after viral infection are varied (25, 32). The most straightforward method is viral replication and shedding leading to eventual cell lysis. A second method of direct oncolysis is the production of cytotoxic viral proteins. Altering production of viral proteins is a target of genetic engineering to improve antitumor efficacy. For example, the adenovirus death protein (ADP) is produced during the normal adenovirus replication cycle to induce host cell death (35); a modified adenovirus designed to overexpress ADP has increased cytolytic activity in a mouse model of lung cancer (36).

A third method of antitumor efficacy is insertion of transgenes into the viral genome, so-called “armed” viruses. An early model of transgene insertion is the HSV thymidine kinase gene, which metabolizes ganciclovir into a toxic byproduct (37). Cells infected with a virus carrying this gene are rapidly lysed in the presence of ganciclovir. Both replication-deficient and replication-competent adenoviral vectors with the HSV thymidine kinase gene have been studied in humans against a number of tumors including mesothelioma, with encouraging results (38–41). Transgenes encoding cytokines such as IL-2 or TNFα to augment immune system response are also utilized (42, 43). With improved methods for oncolytic viruses to specifically target tumor cells, the use of replication-competent viruses armed with transgenes now has become common.

### Tumor Selectivity

**Figure 2:**
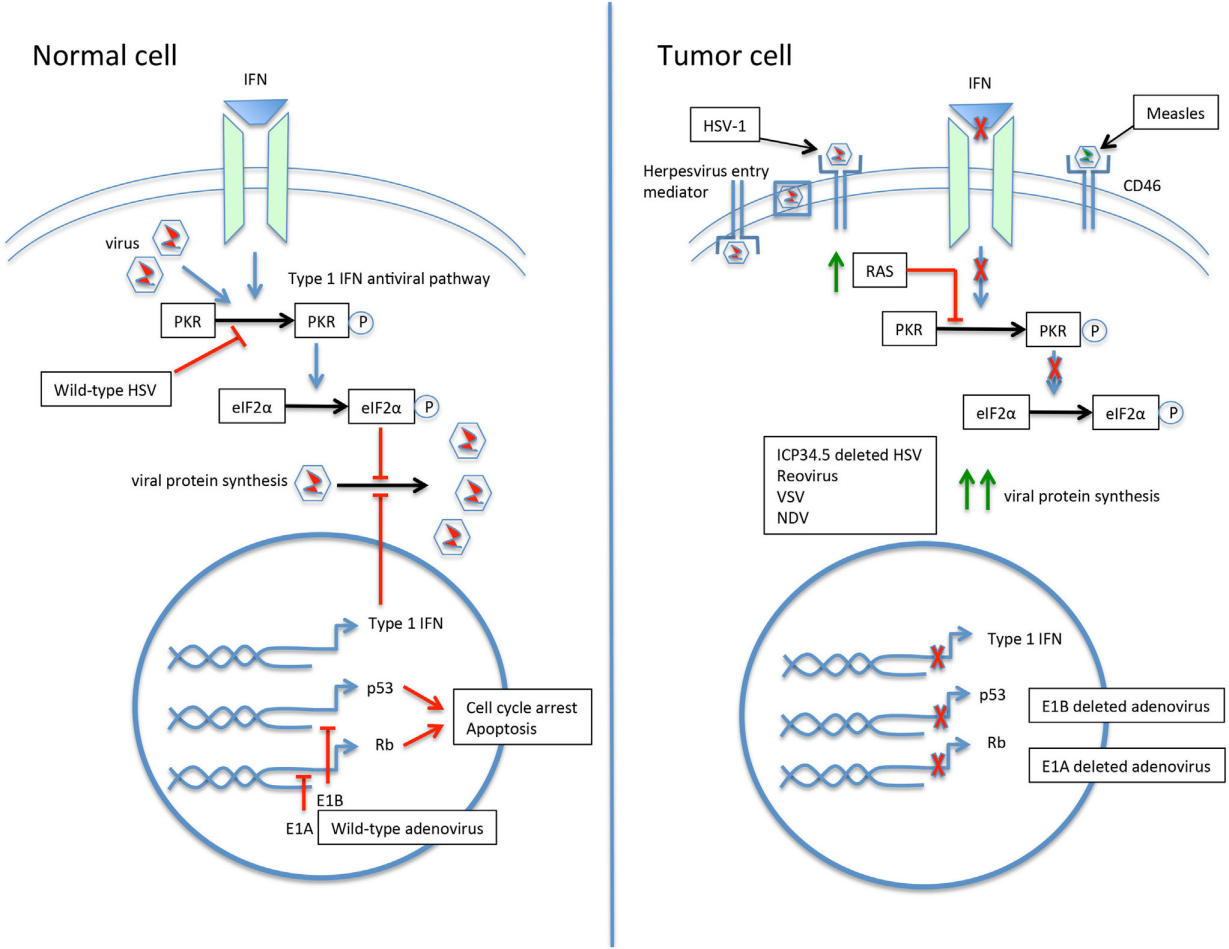
The selective infection of tumor cells by oncolytic viruses. In the normal cell, the response to viral infection involves activation of the type 1 interferon (IFN) and protein kinase R (PKR) pathways, resulting in upregulation of eIF2α and inhibition of viral protein synthesis. The p53 and Rb pathways are also activated. Wild-type viruses are able to inhibit various steps of the antiviral response to allow ongoing replication. For example, the herpes simplex virus (HSV) gene ICP34.5 blocks PKR signaling, and the adenovirus genes E1A and E1B inactivate Rb and p53, respectively. The tumor cell may have a number of acquired defects that allow for preferential infection by oncolytic viruses. An increased expression of cell surface proteins facilitates viral entry, such as herpesvirus entry mediator for HSV type 1 (HSV-1) and CD46 for measles virus. Defective IFN and PKR pathways lead to unimpeded viral protein synthesis. Upregulation of RAS in tumor cells results in PKR pathway inhibition. Modification of viruses can further drive tropism and minimize infection of normal cells. Deletion of the HSV gene ICP34.5 renders the virus unable to inhibit PKR in healthy cells and drives infection toward PKR-deficient tumor cells. Similarly, deletion of the adenovirus E1A or E1B genes leads to preferential infection of p53- and Rb-deficient tumor cells.

Engineering viruses to selectively target tumor cells has proven especially productive. By minimizing infection of and resulting toxicity to normal cells, larger viral doses can be administered and the therapeutic index widened. Mechanisms for engineering viruses for tumor selectivity include modification of the viral coat, exploiting abnormal signaling pathways, insertion of tumor or tissue specific promoters, and partial or entire gene deletions (Figure 2) (26, 34, 44).

Achieving tumor selectivity does not always require a recombinant virus, as a wild-type virus may already exhibit a preference for replicating in tumor cells. This can occur through overexpression of cell surface proteins that facilitate viral entry into the tumor cell (26). Specific viruses have natural tropism for these aberrant proteins, such as HSV-1 for overexpressed herpesvirus entry mediator and nectins on carcinoma cells, measles virus for CD46, and echovirus for an integrin domain on ovarian cancer cells (45–48).

When a natural viral tropism for tumor cell surface proteins is not present, viral coat protein expression can be modified. Ligands unique to the tumor cell surface are identified and the virus engineered for uptake specifically by these ligands (34). This is used in adenoviral vectors by modification of the Ad5 fiber knob domain (49). Another example is a measles virus designed with a surface antibody targeting carcinoma embryonic antigen expressed on adenocarcinoma (50).

Wild-type viruses can also preferentially infect tumor cells by exploiting altered signaling pathways in the tumor cell (44). This illustrates how cellular changes defining malignancy, such as resistance to apoptosis and loss of p53, often overlap with virally induced cellular changes (51, 52). The environment of a tumor cell then may be advantageous by supplying cell processes necessary for viral replication. Two key antiviral pathways present in normal cells are often implicated here—protein kinase R (PKR) and interferon (IFN) signaling (44). A dysfunctional PKR pathway enhances reovirus replication, and defects in the type 1 IFN response potentiate the replication of vesicular stomatitis virus (VSV) and Newcastle disease virus (NDV) (53–55).

Altered tumor cell signaling pathways provide opportunities for viral genetic engineering. Gene deletions can remove viral genes necessary for replication in normal tissue but not required for replication in tumor cells (34). Viral gene products block the normal antiviral response through the PKR, IFN, and p53 pathways. Deletion of these viral genes restores the ability of healthy cells to prevent viral replication, while cancer cells already deficient in the antiviral pathway remain susceptible. HSV-1 modified for deletion of the ICP34.5 gene is such an example. Lacking this gene, the virus no longer blocks PKR signaling in healthy cells, leaving PKR-deficient tumor cells to be preferentially infected (26, 56). Similarly, a modified adenovirus with a gene deletion for the protein E1B no longer inactivates p53. This allows healthy cells to initiate p53-mediated apoptosis prior to viral replication; p53-deficient cancer cells are then selected for viral spread (57).

The goal of most viral gene deletions is to attenuate viral pathogenesis in normal cells. In fact, nearly all oncolytic viruses being studied for clinical use are attenuated in same manner. The first study of a virus modified specifically to improve oncolytic activity, by Martuza et al. in 1991, employed HSV-1 with deletion of the gene encoding the enzyme thymidine kinase (58). This deletion results in attenuated neurovirulence (59).

Just as genes are deleted from the viral genome to increase tumor specificity, insertion of gene promoter regions that are tumor or tissue restricted are frequent additions to achieve the same goal of specificity. This relies on the overexpression of tumor-specific proteins for activation of the promoter region of a gene that is necessary for viral replication and/or cell death. Healthy cells become relative life cycle dead ends for the virus by lacking the proteins needed to activate regulatory viral genes (44). The adenovirus E1A gene has been modified with various gene promoters including an alpha-fetoprotein gene promoter for tumor-specific replication in hepatocellular cancer cells and a prostate-specific antigen gene promoter with tissue-specific replication in prostate cancer (60, 61).

### Immune System Activation

The concepts of viral antitumor efficacy and selectivity can be linked together as the first part of a two-step process necessary for successful oncolytic viral therapy. The initial viral-directed tumor cytotoxicity then must be followed by a sustained antitumor response carried out by the immune system (32). This critical second phase has been recognized for many years (62), although only recently have the mechanisms to make oncolytic viruses a more effective immunotherapy begun to be elucidated (63–65).

Tumor-induced immune tolerance is a critical part of the malignant process. This is accomplished through alteration of the tumor microenvironment by recruitment of immune-inhibitory cells and exclusion of immune-stimulating cells (66). Viral-mediated tumor cell death works to reverse this tolerance by exposing tumor-associated antigens previously restricted from presentation to the immune system, known as neoantigens, and provoking inflammatory cytokine release. Antigen-presenting cells activated by these neoantigens then direct an antitumor response by CD8+ T cells and NK cells (Figure 1) (26).

Prior to arriving at the current paradigm of immune stimulation as an essential part of virotherapy, a major concern was a robust antiviral response limiting the extent of oncolysis (67). In an effort to thwart the immune response, initial murine studies used immunocompromised models to allow adequate viral replication and cytolysis (68). The move to immune-competent models was accompanied by suppression of the immune response, such as dampening T cell response through gene deletions or administering cyclophosphamide prior to viral administration (69, 70). Current approaches aim for a balance between permitting both initial viral replication and the subsequent robust antitumor, and inevitably antiviral, immune response.

Viral genetic engineering now includes modifications to boost immune antitumor activity, often through insertion of cytokine genes. HSV expressing granulocyte macrophage colony-stimulating factor (GM-CSF) increases antigen presentation by dendritic cells and improves tumor reduction of lymphoma in a murine model (56). VSV expressing IFNβ decreased T-regulatory cells, increased CD8+ T cells, and prolonged survival in a murine lung cancer model (65). The HSV-1 protein ICP47 decreases antigen presentation on infected cells, and deletion of this gene augments antitumor effects (56, 71).

The recognition of immune stimulation by oncolytic viruses and the simultaneous development of the immune checkpoint inhibitors raise the possibility of synergy between these distinctive mechanisms of immunotherapy. A number of studies have already been completed in this new area with promising results (72–74). The remarkable success of checkpoint inhibition likely indicates the future role for oncolytic therapy as an adjunct to other more clinically advanced forms of immunotherapy.

### Administration and Safety

The administration of oncolytic viral therapy must account for the setting of metastatic disease that requires a systemic immune response. Intravenous delivery of virus, while having the potential for rapid viral infection at all locations of disease, is problematic for several reasons. An immediate innate humoral immune response may lead to viral inactivation in the bloodstream, prior to infection of tumor cells. In the case of previous environmental exposure or vaccination, antiviral antibodies will provide effective at viral clearance (75–77). Even without preexisting immunity, repeated intravenous administration of virus results in production of antiviral antibody titers that quickly render vascular delivery ineffective (78).

The delivery of oncolytic viruses has predominantly been *via* direct intratumoral (IT) injection. IT administration has its own limitations, most apparent being the requirement of an accessible solid mass. Early viral inactivation by the innate immune system is also an issue with IT injection, although probably to a lesser extent than intravenous therapy (69, 79). Both systemic and direct viral administration must overcome a harsh local tumor environment that limits viral biodistribution (80, 81).

The main advantage of IT administration is ensuring local tumor delivery while also inducing distant tumor responses. This is true in some preclinical models and also in the phase III trial leading to approval of T-VEC (31, 65, 73). With the immune system able to provide a systemic response after local administration, any hypothetical advantage of intravenous delivery is no longer relevant.

Any effective oncolytic therapy needs to take into account effects on surrounding non-cancerous tissues. Of primary concern is the viral infection spreading to healthy cells, given the fact that cancer patients are already immunosuppressed and susceptible to infection. As previously discussed, the concept of selecting and designing a virus with tumor cell selectivity is the key to minimizing toxicity. Additional safety concerns include environmental shedding and reversion to wild-type virus. In general, studies have shown favorable toxicity profiles although perhaps at the expense of efficacy, as the field is now moving toward the use of less attenuated viruses with improved selectivity. A recent review covers safety concerns in more detail (82).

### The Ideal Oncolytic Virus

In addition to optimizing antitumor efficacy, tumor selectivity, and immune system activation, a number of other viral characteristics are taken into account when choosing an oncolytic virus. These include viral genetic stability, non-integrating viruses that cannot incorporate into the host genome, a safety mechanism to inactive the virus after administration, and amenability to high titer production (44). A detailed discussion of these additional factors is beyond the scope of this review.

The ability to non-invasively image viral infectivity is of particular interest. These molecular imaging techniques allow localization of viral replication in tumor or healthy tissue, an important measure of toxicity. The viral dose and route of administration may be correlated with the level of infectivity without the need for repeat tissue biopsies (83). Two viral modifications for molecular imaging are insertion of the gene encoding green fluorescent protein (GFP) or the human sodium iodide symporter (hNIS) protein. The hNIS also offers the potential for radioiodide therapy like that used for ablation of thyroid tissue (84). Many of the mesothelioma studies detailed in the next section utilize addition of these reporter genes, allowing for monitoring of efficacy and toxicity.

With the expanding ability to genetically engineer oncolytic viruses, the use of viruses with multiple modifications is readily available. This is illustrated by T-VEC, an HSV-1 with three separate modifications—deletion of gene ICP34.5 to attenuate neurovirulence, deletion of ICP47 to increase antigen presentation on infected cells, and insertion of the gene for GM-CSF to attract antigen-presenting cells (31, 56, 85–87). This combinatorial approach to maximize efficacy through various mechanisms is now standard, as we will describe with oncolytic virotherapy for mesothelioma.

## Oncolytic Viral Therapy for Mesothelioma

Malignant pleural mesothelioma provides an optimal model for the study of oncolytic virotherapy for several reasons (88). The pleural location is accessible for direct IT injection, the preferred method of administration for most viral platforms. Although distant metastases can occur, complications and death usually stem from local disease spread. Also, limited improvement in outcomes with multimodality therapy lends more urgency to experimental approaches. Given these characteristics, despite being an uncommon malignancy, an extensive amount of preclinical data with oncolytic viruses in mesothelioma models is available. This work has progressed to early phase clinical MPM studies for a number of different viruses (Table 1).

**Table 1:** Human clinical studies of virotherapy for malignant pleural mesothelioma (MPM).

Strain	Modification(s)	Study design	Results
**Adenovirus**			
Ad.HSV*tk* (replication incompetent)	Insertion of thymidine kinase suicide gene	21 patients in single-arm, dose-escalation study received single intrapleural dose followed by ganciclovir (89)	Gene transfer documented in 11 patients, minimal toxicity, no tumor responses
		5 patients given high-dose vector in same method as above study, with addition of systemic steroids (90)	Decreased inflammatory response but no improvement in gene transfer
		Long-term follow-up of 21 patients who received high-dose vector (41)	Good safety profile, two patients lived >6.5 years

Ad.IFNβ (replication incompetent)	Insertion of interferon (IFN)β gene	Phase I dose-escalation study, 7 patients given single intrapleural dose (91)	Clinical response in three patients at 60 days, IFNβ detectable in fluid of eight patients
		Follow-up phase I study, 10 patients given 2 intrapleural doses (92)	Repeated dosing safe, response by CT scan at 60 days in two patients

Adenovirus expressing IFNα2b (replication incompetent)	Insertion of IFNα2b gene	Pilot and feasibility study with 9 patients given 2 intrapleural doses of vector (93)	Five patients with stable disease or tumor regression at 60 days, gene transfer augmented by second dose
		Phase II trial of two intrapleural doses of vector combined with chemotherapy in 40 patients (94)	Partial response in 25%, stable disease in 62.5%, median survival 13 months, six patients lived >2 years

Ad5-D24-GM-CSF (replication competent)	Partial deletion of E1A, insertion of granulocyte macrophage colony-stimulating factor (GM-CSF) gene	20 patients with advanced solid tumors (2 with MPM) given 1 intratumoral dose (64)	47% overall clinical benefit rate, one MPM patient with stable disease

ONCOS-102 (Ad5/3-D24-GM-CSF)	Insertion of Ad3 fiber knob, partial deletion of E1A, insertion of GM-CSF	12 patients with advanced solid tumors (2 with MPM) given multiple intratumoral injections combined with oral cyclophosphamide (95)	Clinical response rate 40% at 3 months, one MPM patient with stable disease, increased PD-L1 in both MPM patients
Ad5/3-D24-GM-CSF		21 patients with advanced tumors (1 with MPM) given one intratumoral and one IV dose, with oral cyclophosphamide (96)	Evidence of efficacy in 13 of 21 patients, MPM patient with stable disease, no grade 4/5 adverse events
**Herpes simplex virus type 1 (HSV-1)**			
HSV-1716 (replication competent)	Deletion of γ_1_34.5 gene	Phase I/IIa study of inoperable MPM with single or multiple intrapleural doses (97)	Pending, expected completion in 2016 (NCT01721018)

**Vaccinia virus**			

VV–IL-2 (replication competent)	Insertion of interleukin-2 gene, deletion of thymidine kinase gene	Small pilot study with six patients receiving multiple intratumoral injections (98)	Well-tolerated, viral gene expression detected for up to 3 weeks after administration, no tumor responses

JX-594 (replication competent)	Deletion of thymidine kinase gene, insertion of GM-CSF gene	Phase I trial, 23 patients with metastatic solid tumors (1 MPM patient), given single IV dose (99)	No dose-limiting toxicities, MPM patient with partial response for >10 weeks
**Measles**			
Measles virus (MV)–NIS (replication competent)	Edmonston strain with insertion of NIS gene	Phase I trial enrolling patients with MPM confined to single pleural cavity, given q28 days for up to six cycles (100)	Pending, currently enrolling patients (NCT01503177)
**Newcastle disease virus**			
PV701 (replication competent)	Naturally attenuated, non-recombinant	Phase I trail of 79 patients with advanced solid malignancies (2 with mesothelioma), virus given intravenously at various doses and intervals (101)	9 patients with objective responses, 1 *peritoneal* mesothelioma patient with 35% tumor reduction and received 30 total doses, 1 MPM patient with progressive disease
**Reovirus**			
Type 3 Dearing strain (replication competent)	Wild-type, non-recombinant	Phase 1 trial in 25 patients with advanced malignancy (1 MPM patient), given IV q3 weeks at escalating doses, combined with docetaxel (102)	Disease control rate 88%, MPM patient with minor response

Studies using replication-incompetent viruses are most accurately classified as gene therapy with a viral vector rather than oncolytic virotherapy, since “oncolytic” implies active viral replication. In the context of cancer, gene therapy is the transfer of genetic material to induce tumor cell death, as defined by Sterman (88). This can be accomplished in a number of ways, and oncolytic virotherapy is a subtype of gene therapy using actively replicating viruses. Given the significant overlap, studies of both replication-competent and replication-incompetent viruses are reviewed here.

### Adenovirus

A non-enveloped virus with a linear, double-stranded DNA genome, adenovirus is one of the most extensively studied oncolytic viruses, rivaled only by HSV. A moderately sized genome of ~38 kilo base pairs (kb) allows for multiple modifications (26, 32). Other favorable characteristics are a stable genome, non-integration, and high-titer production. Most humans are exposed and asymptomatic upon infection, although susceptible individuals can develop upper respiratory symptoms or conjunctivitis (33).

Studies with oncolytic adenovirus have advanced to many early phase human trials including prostate, pancreatic, and colorectal carcinomas (60, 103, 104). Notably, a phase III trial for head and neck squamous cell carcinoma conducted in China combined an E1B-deleted adenovirus (H101) with chemotherapy (29). This led to approval in China of H101 for treatment of nasopharyngeal carcinoma in combination with chemotherapy.

Adenovirus for mesothelioma includes both preclinical and human studies. The first *in vitro* studies out of the University of Pennsylvania focused on gene therapy with the HSV-thymidine kinase suicide gene inserted into a replication-deficient adenoviral vector (Ad.HSV*tk*) (105, 106). This same adenovirus then proved successful in animal models (107, 108). For example, Elshami and colleagues in 1996 used a rat model of MPM to intratumorally administer Ad.HSV*tk* followed by systemic ganciclovir, which is metabolized into toxic byproducts by the HSV*tk* gene product (109). The experimental rats showed tumor regression at 20 days (average tumor weight 0.6 vs. 5.4 g) and improved mean survival (34 vs. 26 days) compared to controls.

Replication-deficient adenovirus has also been studied as a vector for cytokine gene therapy to counter the immune tolerance characteristic of mesothelioma (88). After passive immunotherapy with intrapleural or systemic delivery of IL-2, IFNα, and IFNγ for mesothelioma met with some success in phase I/II trials, administration of cytokines *via* gene therapy was proposed to improve efficacy (110–112). In several murine experiments, IT injection of an adenovirus with insertion of the IFNγ gene resulted in tumor regression and a CD8+ T cell-mediated response (113, 114). By using a different mechanism to induce antitumor immunity, a replication-deficient adenovirus engineered to express the costimulatory molecule CD40L showed regression of both directly injected and distant tumors, indicative of a systemic immune response (115).

More recently, a series of preclinical studies using conditionally replicating adenoviruses (CRAds), replication-competent oncolytic viruses with modifications to improve tumor selectivity, have shown antimesothelioma activity. Instead of the E1 gene being deleted to produce replication-incompetent viruses, the gene is placed under control of tumor-specific promoters. An *in vitro* study inserted a midkine promoter overexpressed in tumor cells and demonstrated effective oncolysis in human MPM cell lines (116). *In vivo* studies with murine models have used a number of CRAd modifications—promoters linked to E1 gene expression, viral capsid alterations, and insertion of GFP for viral imaging (117, 118). For example, Watanabe and colleagues in 2010 used an adenovirus engineered to express a telomerase-driven promoter linked to the E1 gene (119). This was co-administered with a replication-incompetent adenovirus with insertion of the heparanase gene to improve viral penetration through the dense extracellular matrix. The study showed significant tumor regression compared to controls as well as improved survival.

Human studies using adenoviral vectors began relatively quickly following preclinical experiments. Consecutive phase I dose-escalation studies used the replication-incompetent Ad.HSV*tk* gene therapy followed by ganciclovir (89, 90, 120). The response rate was low, with 1 of 26 patients having evidence of tumor regression. Seventeen patients had evidence of IT gene transfer on repeat pleural biopsy, although this was limited to the outermost tumor layers. A follow-up study of 21 patients who received “high-dose” therapy reported a good safety profile and 2 patients surviving >6.5 years (41). Although these studies proved to be safe, the low response rate indicated a need for improved gene transfer within the tumor and a more robust antitumor immune response.

Focusing on stimulating an immune response rather than delivering a suicide gene, several human trials have been completed using an adenoviral vector for gene transfer of IFNβ (Ad.IFNβ). These studies, like those for Ad.HSV*tk*, were with replication-incompetent virus. The initial phase I dose-escalation trial enrolled seven patients with MPM and three patients with metastatic pleural effusions due to other malignancies, administering a single intrapleural dose of Ad.IFNβ (91). At 60 days, three patients with MPM had a clinical response and four patients had progressive disease. IFNβ was detectable in the pleural fluid of eight patients, indicating successful gene transfer.

A follow-up phase I trial with Ad.IFNβ evaluated giving a second intrapleural dose (92). Repeated administration was safe, and 2 of 10 MPM patients had a clinical response by CT scan at 2 months. This lack of improvement in response with repeat dosing was likely from rapid development of neutralizing antibodies, as humoral immune responses were consistently detected but not the cellular responses more essential to antitumor immunity. Although these two IFNβ gene therapy trials were encouraging for stimulating an immune response, further dose modifications or combination therapy are needed to have a more significant impact on outcome.

When Ad.IFNβ was no longer commercially available, a subsequent phase I gene therapy study by Sterman and colleagues was completed using replication-incompetent adenovirus expressing IFNα2b (Ad.IFNα2b) (93). Clinical responses were encouraging, with five of nine patients having stable disease or tumor regression at 60 days. This led to a phase II trial combining Ad.IFNα2b with chemotherapy in 40 patients (94). Patients received two intrapleural doses of Ad.IFNα2b on days 1 and 4, followed by chemotherapy on day 15 with standard of care pemetrexed/platinum doublet for chemotherapy naïve patients (first-line treatment, 18 patients). If this was second-line chemotherapy, gemcitabine or pemetrexed was given (22 patients). Partial responses were observed in 25% of patients and stable disease in 62.5%. Although the median overall survival of 13 months was not significantly improved from standard treatments, six patients lived more than 24 months. Based on these results, a randomized phase III trial is planned.

Studies using replication-competent, oncolytic adenoviruses for MPM are scarce. A study by Cerullo and colleagues evaluated an oncolytic adenovirus (Ad5-D24-GM-CSF) modified for tumor selectivity and with insertion of a transgene for GM-CSF to augment immune response (64, 121). The virus was administered once intratumorally to 20 patients with advanced solid cancers, including 2 patients with MPM. Both patients had received prior chemotherapy. Reflecting the clinical benefit rate of 47% among all patients, one case of MPM had stable disease and lived over 1 year; the other case had progressive disease and lived about 100 days. No serious adverse events occurred.

A phase I study published in 2016 used Ad5/3-D24-GM-CSF in patients with advanced solid tumors (95). Twelve patients were enrolled including two with MPM. Multiple IT injections were given along with oral cyclophosphamide. Clinical response rate at 3 months was 40%; one MPM patient had stable disease, and the other had progressive disease. Tumor-infiltrating leukocytes increased following treatment in 11 of 12 patients. Interestingly, both patients with MPM showed increased PD-L1 expression posttreatment, relevant for potential future combination studies with immune checkpoint inhibitors.

### HSV Type 1

An enveloped, double-stranded DNA virus, HSV-1 has a large 152 kb genome. About 30 kb of the genome is non-essential, which allows space for insertion of transgenes (33). The human pathogenesis of HSV-1 causes oral and genital lesions, latent infection in peripheral nerves, and less frequently CNS complications. This natural tropism for neuronal tissue led to early studies on brain tumors and also necessitates viral gene deletions to attenuate neurotoxicity in all oncolytic experiments using HSV-1 (58, 59, 122). Recombinant HSV-1 has been studied in a number of malignancies including colorectal, pancreatic carcinoma, and melanoma (31, 123, 124). In fact the only FDA-approved oncolytic virus, T-VEC for melanoma, is a modified HSV-1.

A preclinical study by Kucharczuk and colleagues in 1997 evaluated the replication-competent, neuroattenuated HSV-1716 as oncolytic virotherapy for mesothelioma (125). Neuroattenuation was achieved by deletion of both γ_1_34.5 genes encoding the protein ICP34.5. The virus efficiently replicated in and lysed human mesothelioma cells *in vitro*. The same human mesothelioma cell line was then used to establish intraperitoneal tumors in immunodeficient mice. Fourteen days later, the mice were given HSV-1716 by intraperitoneal injection, resulting in decreased tumor burden and increased survival compared to controls. No viral dissemination was detected in non-tumor tissue.

Another preclinical study evaluated three different replication-competent, oncolytic herpesviruses: G207, NV1020, and NV1066 (126). G207 has both γ_1_34.5 genes deleted along with inactivation of the ICP6 gene for additional attenuation in non-replicating tissues. NV1020, originally designed as an HSV vaccine, has deletions encoding the genes ICP0, ICP4, latency-associated transcripts, one copy of γ_1_34.5, and UL24, all resulting in loss of virulence. NV1066 has single copy deletions of ICP0, ICP4, and γ_1_34.5, plus the addition of GFP for viral imaging. Each virus was tested against 11 different MPM cell lines *in vitro*, including each histologic subtype of MPM—epithelioid, sarcomatoid, biphasic, and mixed. All three viruses were cytotoxic to each cell line, even at low multiplicities of infection (the ratio of viral particles to tumor cells). A murine model of MPM treated with NV1066 decreased tumor burden and increased survival.

Adusumilli and colleagues at Memorial Sloan-Kettering Cancer Center did several additional elegant *in vitro* studies combining NV1066 with other therapies. The first study evaluated viral replication and cytotoxicity in 10 human MPM cell lines infected with NV1066 with or without cisplatin (127). The combination proved synergistic, at least partly attributable to cisplatin-induced DNA damage upregulating the protein GADD34 that in turn potentiates replication and cytotoxicity of the mutant HSV-1.

The second study combined NV1066 and radiation therapy in multiple human MPM cell lines and found synergistic or additive effects depending on the cell line, based on the same mechanism of GADD34 upregulation (128). A murine flank tumor model demonstrated reduced tumor growth with the combination compared to controls or either therapy alone. Importantly, in both of these studies, cytotoxicity was maintained with dose reductions, which may allow for decreased toxicity if such combination therapy advances to further trials.

Human studies of oncolytic herpesviruses for mesothelioma have not been completed. An ongoing phase I/IIa study of HSV-1716 for inoperable MPM began recruiting in 2012. The virus is delivered into the pleural cavity in single or multiple doses, with safety as primary outcome and efficacy as secondary outcome. Study completion is expected in 2016 (97).

### Vaccinia Virus

An enveloped, double-stranded DNA virus in the poxvirus family, vaccinia virus has a large ~190 kb genome that facilitates insertion and deletion modifications to improve oncolytic efficacy. Vaccinia replicates in the cytoplasm, posing no risk for host genome integration. An attenuated vaccinia virus was used to eradicate smallpox. Pathogenesis of the wild-type virus in immunocompetent humans is limited to a mild viral syndrome of fever, rash, and myalgias (26, 33).

Vaccinia viruses have been studied in a number of solid tumors in humans including breast, melanoma, and prostate (129–131). Some of these recombinant viruses are described as vaccines, since the objective is stimulation of an antitumor immune response rather than active viral replication causing oncolysis. JX-594, the most clinically advanced oncolytic vaccinia virus, has deletion of the thymidine kinase gene and an inserted transgene to express GM-CSF (132). A phase III trial combining JX-594 with sorafenib for treatment of hepatocellular carcinoma is now recruiting patients (133, 134).

Preclinical and human studies have evaluated treatment of mesothelioma with vaccinia viruses. The replication-competent vaccinia virus GLV-1h68 has deletions of the hemagglutinin and thymidine kinase genes for attenuation and insertion of three transgenes including GFP for viral imaging (135). GLV-1h68 successfully replicated in and lysed multiple MPM cell lines *in vitro* (136). The same study then established a murine model of MPM followed by intrapleural delivery of the virus that resulted in decreased tumor burden and increased survival. The GLV-1h153 virus, a modification of the parent virus GLV-1h68 by insertion of the hNIS gene, proved similarly effective for *in vitro* and murine models with the addition of radioiodine-based imaging for viral infection (137).

A study by Acuna and colleagues in 2014 evaluated vaccinia virus as adjuvant therapy following surgery in a murine model of malignant peritoneal mesothelioma (138). An oncolytic strain with deletions in thymidine kinase and vaccinia growth factor genes for tumor selectivity was used, the double-deleted vaccinia virus (vvDD). A single intraperitoneal dose of vvDD prolonged survival compared to controls. When combined with incomplete cytoreductive surgery, survival was not significantly prolonged compared to either vvDD alone or surgery alone. This led the investigators to propose that the effectiveness of vvDD as an adjuvant therapy following surgery may be limited to microscopic disease after complete surgical resection, although further studies have not yet been completed to confirm this hypothesis.

Human studies with vaccinia virus from MPM are limited. A small pilot study published in 2000 used a vaccinia virus with insertion of the IL-2 gene into the thymidine kinase gene region, a replication-competent virus with tumor cell selectivity (98). Six patients received multiple IT injections. Treatment was well tolerated, and viral gene expression was detected for up to 3 weeks after injection despite the development of antivaccinia antibodies; however, no tumor responses were seen.

An early phase I trial with the oncolytic JX-594 vaccinia virus enrolled 23 patients with metastatic solid tumors, including 1 patient with MPM (99). In contrast to IT or intrapleural administration in nearly every other study, this virus was given intravenously as vaccinia has natural mechanisms to prevent inactivation in blood (139, 140). Following a single intravenous administration, the patient with MPM had a partial response for greater than 10 weeks.

### Measles Virus

An enveloped RNA virus with a small ~15 kb genome, measles virus (MV) is a well-known human pathogen that occasionally causes serious illness in non-vaccinated individuals. The attenuated Edmonston strain is used for oncolytic virotherapy given its proven safety profile and also natural tumor specificity due to the upregulation of CD46 on tumor cell surface that the virus uses for cellular uptake (26, 47, 48). Other favorable characteristics of MV are a stable genome and cytoplasmic replication.

Oncolytic measles viruses have been studied in both solid and hematologic malignancies (141, 142). The most visible success thus far is a preliminary report from the Mayo Clinic of two relapsed, refractory myeloma patients given attenuated MV intravenously, with one patient achieving a complete remission lasting 9 months (143). This phase I/II study for myeloma patients is continuing to enroll patients (144).

Several preclinical studies with MV in MPM have been completed. The first *in vitro* experiment used the live attenuated Schwartz strain to evaluate oncolytic activity and immune response against human mesothelioma cells and normal mesothelial cells (145). The mesothelioma cells were more susceptible to infection and viral-induced cell death than the mesothelial cells, attributed to increased CD46 expression on the cancerous cells. Dendritic cells phagocytized the apoptotic MV-infected mesothelioma cells, resulting in dendritic cell maturation and priming of CD8+ T cells. Although *in vitro*, these results were encouraging for MV-stimulating antitumor immunogenicity.

Li and colleagues used a murine model of mesothelioma to study Edmonston strain MV with insertion of the IFNβ and NIS genes (MV–mIFNβ–NIS) (146). After confirming infectivity and replication of the virus *in vitro*, mice were injected subcutaneously in the flank with mesothelioma cells. After tumors grew to 5 mm, different MVs were injected intratumorally. Tumors injected with MV–mIFNβ had increased immune cell infiltration and decreased angiogenesis compared to tumors injected with the parent MV without mIFNβ expression. These pathological findings correlated with median survival, which increased from 20 days for control mice, to 45 days for mice receiving MV without mIFNβ, to 65 days with MV-mIFNβ. A peritoneal mesothelioma mouse model showed similar improvements in survival for each virus. In addition, the NIS gene facilitated non-invasive radioiodine imaging.

A more recent *in vitro* study published in 2015 evaluated the mechanism of MV for tumor cell selectivity. Twenty-two MPM cell lines were tested for infectivity and replication of MV, along with four healthy cell lines. Interestingly, the amount of CD46 expression did not predict for MV infectivity, contrary to previous assumptions. A better correlate for sensitivity to MV was the ability to mount a complete type 1 IFN response. Cell lines unable to generate or respond to IFNα or IFNβ, the case for 70% of the MPM lines, were more susceptible to MV infection (147). These data have implications for predicting response in future studies of MPM to oncolytic MV.

No human studies of MV for mesothelioma have yet to be completed. A phase I trial using the attenuated Edmonston strain with insertion of the NIS gene (MV–NIS) is currently enrolling patients with MPM confined to a single pleural cavity (100). The virus is administered intrapleurally once every 28 days for up to six cycles. Primary and secondary objectives are maximum tolerated dose, safety, and toxicity; tertiary objectives are measurements of viral activity, immune response, and efficacy.

### Other Oncolytic Viruses for Mesothelioma

Adenovirus, HSV-1, vaccinia virus, and MV are the most extensively studied virotherapy vectors for mesothelioma. A more limited number of studies have evaluated additional viruses including VSV, NDV, reovirus, and Sendai virus.

An RNA virus in the Rhabdoviridae family, VSV has no known pathogenesis in humans. Exposure is possible in those working with livestock or mice; otherwise the general population is immune naïve. This lack of pre-formed immune response is an advantage when introducing VSV as an oncolytic virus (65). VSV displays natural tumor selectivity *via* induction of the antiviral type 1 IFN pathway. In healthy cells with intact IFN signaling, this prevents viral replication, whereas tumor cells with a defective IFN response allow viral infection to proceed unimpeded (148).

Recombinant VSV engineered to express IFNβ (VSV–IFNβ) augments both the antiviral defense in healthy tissue and the immune response against tumor cells. Several preclinical studies have evaluated VSV–IFNβ against mesothelioma. A murine model of subcutaneous and intraperitoneal tumors injected with VSV–IFNβ showed reduced tumor growth and increased survival compared to controls (149). Safety was also enhanced, with less neurotoxicity with mouse IFNβ.

A second study looked at mesothelioma cell lines *in vitro* and correlated cytotoxicity from VSV–IFNβ with the extent of IFN responsiveness (150). Partial responsiveness, measured by upregulation of PKR and other elements after viral infection, led to resistance to cytolysis. Conversely, downregulation of p48 and PKR caused sensitivity to the virus. The authors proposed that testing tumor cells for IFN responsiveness might provide a predictive marker for this virotherapy.

NDV is an RNA avian paramyxovirus that causes serious disease in fowl but only mild disease in humans. Similar to VSV, the tumor specificity of NDV is dictated through a defective type I IFN pathway in tumor cells (151). A preclinical study in mesothelioma with NDV engineered to express GFP showed effective oncolysis against multiple mesothelioma cell lines *in vitro* (152). An orthotopic model of MPM in mice was then treated with either single or multiple intrapleural doses of NDV. Animals receiving multiple treatments had decreased tumor burden, measured by bioluminescence imaging of GFP. Survival was longest in those receiving multiple treatments and shortest in the control group.

A phase I trial using a replication-competent NDV enrolled 79 patients with advanced solid malignancies, including 2 cases of mesothelioma (101). The virus was administered intravenously at various dose levels and intervals. Of the 9 patients with objective tumor responses, 1 patient with peritoneal mesothelioma received over 30 doses of virus with a 35% tumor reduction, improved performance status, and no cumulative toxicity. A posttreatment tumor biopsy showed active NDV replication. Despite this encouraging result, no further human studies with NDV for mesothelioma have been completed.

Reovirus and Sendai virus are two additional RNA viruses that have been studied in combination with chemotherapy for mesothelioma. In a murine model of MPM, Sendai virus with cisplatin showed synergistic effects (153). A phase I trial evaluated intravenous reovirus plus docetaxel in 25 patients with advanced cancer (102). The one patient with mesothelioma had a minor response.

### The Future of Oncolytic Virotherapy for Mesothelioma

The current paradigm for treatment of MPM emphasizes a multimodality approaching with surgery, radiation, or chemotherapy. Most studies of oncolytic therapy for MPM have been as monotherapy, necessary to confirm viral activity, dosing, and safety in preclinical and early-phase human trials. However, a number of studies have successfully combined virotherapy for mesothelioma with chemotherapy (94, 127, 154), radiation (128), and surgery (138, 155). Given the documented safety but overall limited efficacy thus far when administered as monotherapy, future studies will likely use oncolytic viruses as an adjuvant to more established therapy (156).

Combining the immune checkpoint inhibitors with oncolytic viruses is of exceptional interest given the synergistic mechanisms of immune activation. In fact since the recent approval of the oncolytic virus T-VEC for melanoma, a study has already shown improved efficacy with T-VEC when given with the CTLA-4 inhibitor ipilimumab (72). A study by Patel and colleagues finding increased tumor expression of PD-L1 after treatment with VSV–IFNβ in a murine model of non-small-cell lung cancer indicates the potential of PD-1/PD-L1 agents to increase efficacy (65). Early phase trials with immune checkpoint inhibitors for mesothelioma are in process, with promising preliminary results (157).

The ability to molecularly engineer recombinant viruses to improve safety and efficacy has led to rapid advances in oncolytic virotherapy over the last several decades. Preclinical work for mesothelioma is now starting to move into more significant human studies with several clinical trials currently recruiting patients (97, 100). As the field continues to develop, more studies are needed to determine how oncolytic virotherapy is best utilized alongside current treatment for mesothelioma.

## Author Contributions

DP wrote the first draft of this article in consultation with RK. RK conceived and edited the article, as well as drafting the format.

## Conflict of Interest Statement

The authors declare that the research was conducted in the absence of any commercial or financial relationships that could be construed as a potential conflict of interest.
